# Assessing longitudinal patterns of depressive symptoms and the influence of symptom trajectories on HIV pre‐exposure prophylaxis adherence among adolescent girls in the HPTN 082 randomized controlled trial

**DOI:** 10.1002/jia2.25731

**Published:** 2021-06-24

**Authors:** Jennifer Velloza, Sybil Hosek, Deborah Donnell, Peter L Anderson, Mike Chirenje, Nyaradzo Mgodi, Linda‐Gail Bekker, Sinead Delany‐Moretlwe, Connie Celum, HPTN 082 study group

**Affiliations:** ^1^ University of Washington Department of Global Health Seattle WA USA; ^2^ Stroger Hospital of Cook County Department of Psychiatry Chicago IL USA; ^3^ Vaccine and Infectious Disease Division Fred Hutchinson Cancer Research Center Seattle WA USA; ^4^ Department of Pharmaceutical Sciences University of Colorado Aurora CO USA; ^5^ University of Zimbabwe College of Health Sciences Clinical Trials Research Centre Harare Zimbabwe; ^6^ The Desmond Tutu HIV Centre University of Cape Town Cape Town South Africa; ^7^ Wits Reproductive Health & HIV Institute (Wits RHI) Faculty of Health Sciences University of the Witwatersrand Johannesburg South Africa; ^8^ Department of Medicine University of Washington Seattle WA USA

**Keywords:** depression, psychosocial, HIV, pre‐exposure prophylaxis, Africa, women

## Abstract

**Introduction:**

African adolescent girls and young women (AGYW) eligible for HIV pre‐exposure prophylaxis (PrEP) experience high levels of depressive symptoms. Depression can reduce PrEP adherence among adults, although analyses have considered depression as a time‐varying exposure rather than modelling distinct patterns of symptoms. The association between depressive symptoms and PrEP adherence has not been explored for AGYW. To address these gaps, we sought to understand depressive symptom trajectories among African AGYW initiating PrEP and the impact of time‐varying depressive symptoms and symptom trajectories on PrEP adherence.

**Methods:**

HPTN 082 was an open‐label PrEP study among AGYW (ages 16 to 24) in Zimbabwe and South Africa from 2016 to 2018. Depressive symptoms were measured at enrolment and Weeks 13, 26 and 52, using the 10‐item Center for Epidemiologic Studies scale; a score ≥10 is indicative of elevated depressive symptoms. PrEP adherence was defined as any detectable tenofovir diphosphate (TFV‐DP) levels. Group‐based trajectory modelling was used to model longitudinal patterns of depressive symptoms. We assessed psychosocial and behavioural predictors of depressive symptom trajectory membership (e.g. PrEP stigma, intimate partner violence [IPV], sexual behaviour). We modelled associations between (1) group trajectory membership and PrEP adherence at Week 52 and (2) time‐varying depressive symptoms and PrEP adherence through follow‐up.

**Results:**

At enrolment, 179 (41.9%) participants had elevated depressive symptoms. Group‐based trajectory models revealed persistent elevated depressive symptoms in 48.5%, declining symptoms in 9.4% and no consistent or mild depressive symptoms in 43.3%. AGYW who engaged in transactional sex, reported IPV, or had traumatic stress symptoms were more likely to be assigned to the persistent elevated symptom group compared with the consistent no/mild symptom group (Wald test *p*‐value all <0.01). Participants assigned to the persistent elevated depressive symptom trajectory had a significantly lower risk of detectable TFV‐DP at Week 52 than those in the no/mild symptom trajectory (adjusted prevalence ratio = 0.89; 95% CI: 0.80 to 0.98). Elevated depressive symptoms were significantly inversely associated with PrEP use throughout follow‐up (adjusted relative risk = 0.73; 95% CI = 0.53 to 0.99).

**Conclusions:**

Persistent depressive symptoms were common among African AGYW seeking PrEP. Integration of depressive symptom screening and treatment into PrEP programmes may improve PrEP effectiveness among African women.

## 1 Introduction

African adolescent girls and young women (AGYW) ages 15 to 24 face high rates of HIV. Recent trials among AGYW ages 18 to 25 years report HIV incidence rates as high as 7 per 100 person‐years [1, 2, 3]. HIV pre‐exposure prophylaxis (PrEP) is a highly efficacious HIV prevention strategy being scaled up for high‐risk populations including AGYW in HIV endemic settings [4, 5, 6, 7, 8, 9]. Demonstration projects with adolescents report high initial PrEP uptake but declines in adherence and continuation in real‐world settings [10, 11, 12]. Lower PrEP adherence over time among AGYW is associated with complex psychosocial and behavioural factors such as lack of social support, intimate partner violence (IPV) and PrEP stigma [13, 14, 15, 16].

AGYW at risk of HIV also face high levels of depression [15, 16]. Studies in sub‐Saharan African cohorts found that approximately 20% to 50% of young women initiating HIV prevention services have mild‐to‐moderate depressive symptoms [14, 16, 17]. Individual and interpersonal (e.g. IPV, stigma, low social support) and community‐level and structural (e.g., food insecurity, gender inequality) factors, often acting together, can cause elevated risk of depression and HIV acquisition among women [17, 18, 19]. PrEP trials with South African, Kenyan and Ugandan women estimated that those with depressive symptoms had 25% to 27% lower PrEP adherence than those without symptoms and this relationship persists after adjusting for IPV, social support and stigma [14, 16]. Negative relationships between depressive symptoms and PrEP adherence have also been reported among young men who have sex with men and transgender women, suggesting that these findings are generalizable across youth at risk for HIV [20, 21]. The HIV treatment and contraceptive fields also consistently report negative associations between depressive symptoms and medication adherence among young women in the United States and Africa [22, 23, 24, 25, 26, 27].

While prior research has explored associations between depressive symptoms and PrEP adherence in adult women, gaps remain in understanding distinct patterns of depressive symptoms over time and whether various trajectories of depressive symptom severity differentially impact PrEP use among AGYW. Depressive symptoms could change dynamically over time and recur, particularly among AGYW [17, 28], and a nuanced view of depressive symptom patterns and how different patterns influence PrEP adherence is needed. Several studies have explored depressive symptom trajectories among adults in South Africa and the United States [29, 30, 31], but only one analysis to date modelled symptom trajectories in AGYW and was conducted in the United States [28]. These studies document differences between participants with distinct depressive symptom trajectories (e.g. declining, persistent, low or resurging symptoms) but did not explore associations between trajectory membership and HIV‐related outcomes. To address these gaps, we analysed data on depressive symptoms and PrEP adherence from a clinical trial of AGYW initiating PrEP in South Africa and Zimbabwe. Our objectives were to (1) model depressive symptom trajectories among African AGYW; (2) assess baseline characteristics associated with trajectory membership and (3) estimate associations between time‐varying depressive symptoms and depressive symptom trajectories on PrEP adherence. Our findings could guide future PrEP delivery efforts for African AGYW to improve both mental health and PrEP adherence outcomes in this population.

## 2 Methods

### 2.1 Study design and participants

The HIV Prevention Trials Network (HPTN) 082 study was a randomized, open‐label trial of adherence interventions among African AGYW initiating daily oral emtricitabine tenofovir (FTC/TDF)‐based PrEP [32]. Eligible AGYW were HIV‐negative, between 16 and 25 years old, sexually active and at high risk of HIV [33], literate in one or more study languages and residing in Johannesburg, Cape Town or Harare. Enrolment and follow‐up occurred from 2016 to 2018.

Participants were offered daily oral PrEP at enrolment. Those who accepted PrEP were randomized in 1:1 ratio to either the control group with a standard PrEP adherence package (counselling sessions, weekly SMS reminders, monthly in‐person adherence support clubs) or the intervention group (the standard package plus counselling based on PrEP drug levels, called “drug‐level feedback counselling”). Follow‐up visits took place at Weeks 4, 8, 13, 26, 39 and 52 post‐enrolment. Participants received HIV testing, counselling and PrEP refills at all visits.

### 2.2 Data collection

Sociodemographic data were collected at enrolment, including age, marital status and education. At enrolment and follow‐up visits, participants completed computer‐assisted self‐interviewing (CASI) surveys to provide data on sexual behaviour (number of sex acts, condom use, number of partners, transactional sex), depressive symptoms, alcohol use, social support, stigma related to HIV and PrEP, IPV and post‐traumatic stress.

Depressive symptoms were measured using the 10‐item Center for Epidemiologic Studies (CES‐D) scale at enrolment and Weeks 13, 26 and 52 [34]. Sum scores were calculated (range 0 to 30; Cronbach’s alpha: 0.76), and a score ≥10 was indicative of elevated depressive symptoms [34]. This cut‐off was previously used with African adolescents, enabling comparisons across studies, and has good sensitivity and sensitivity in several study languages (e.g. isiXhosa, isiZulu) [34, 35, 36, 37, 38]. Participants were provided with referrals for mental health care with a physician, psychologist or social worker based on participant request and/or clinician discretion.

Other psychosocial and behavioural variables were measured at enrolment and Weeks 13, 26 and 52. Alcohol use was measured using the three‐item AUDIT‐C scale with a score ≥3 indicative of alcohol misuse [39]. Social support was measured with two items assessing social support from adults, adapted from prior work with young African women (Table S1) [40, 41]. Responses were scored from 0 to 2 and summed, with higher scores indicating higher support (range: 0 to 4). Internalized stigma related to HIV and PrEP use was assessed with six items (Table S2), adapted from PrEP stigma questions for adults and key findings on PrEP barriers among adolescents [42, 43, 44]. Items were scored from 0 to 4 and summed, with a higher sum score indicating greater internalized stigma (range: 0 to 24; Cronbach’s alpha: 0.89). IPV was assessed with four items (Table S3), developed from the World Health Organization’s operational IPV definitions [45]. Participants were considered to have experienced IPV if they answered “yes” to at least one item. Traumatic stress was assessed with the four‐item posttraumatic stress disorder (PTSD) Checklist for the DSM‐5 (PCL‐5) [46]. An answer of “yes” to any item was considered indicative of PTSD symptoms.

The primary outcome was PrEP adherence measured using intracellular tenofovir‐diphosphate levels (TFV‐DP) in dried blood spots (DBS) collected at Week 13, 26 and 52. TFV‐DP provides an estimate of cumulative PrEP dosing and has been validated among women and men who have sex with men in directly observed therapy studies [47]. For our primary analyses, PrEP adherence was defined as detectable versus undetectable TFV‐DP levels (≥16 fmol/punch as a limit of detection), which represents any PrEP use since the prior visit [47, 48]. We also conducted descriptive analyses of high PrEP adherence, defined as TFV‐DP levels ≥700 fmol/punch, which represents consistent dosing (≥4 PrEP doses per week) [47, 48].

### 2.3 Group‐based trajectory modelling analysis

Group‐based trajectory modelling was used to identify patterns of depressive symptoms during follow‐up. Models estimate the proportion of a population belonging to different trajectories and calculate the posterior probability of an individual belonging to an assigned trajectory given observed data. Based on similar analyses conducted with populations in the United States, we hypothesized finding depressive symptom trajectories corresponding to low symptoms, recurring symptoms, declining symptoms, and/or persistent elevated symptoms. Depressive symptoms were modelled as a binary variable (CES‐D score </≥10), using a binomial distribution.

The selection of the best‐fitting trajectory model was a two‐step process. First, we identified the optimal number of trajectory groups by fitting unconditional trajectory models for depressive symptoms with 2 to 6 trajectories. This range of trajectories was decided *a priori* to maximize interpretability and public health utility. All trajectory groups were modelled using cubic polynomial terms in this step [49]. We compared models using Bayesian Information Criterion (BIC) and average posterior probabilities, with average probabilities ≥0.70 indicating good fit [49]. Second, we identified the optimal function form for each trajectory. We compared linear, quadratic and cubic terms for each trajectory and selected the functional form based on BIC values, posterior probabilities and visual inspection. We also conducted an exploratory group‐based trajectory model analysis using continuous CES‐D score following the same two‐step procedure with a censored normal distribution.

After determining optimal trajectory number and form, we subsequently assessed psychosocial and behavioural characteristics associated with depressive symptom trajectory membership. Individuals were assigned to trajectories with the highest posterior probability [50, 51]. We added covariates for study arm, site, age, education, sexual behaviour, alcohol use, social support, stigma, any IPV and PTSD symptoms. We only included baseline values of these variables to ensure the temporality of our associations. We used Wald and Wilcoxon rank sum tests to compare baseline characteristics across trajectories.

Group‐based trajectory models assume missing data are missing completely at random [49]. We compared participants with missing and complete depressive symptom data. In a sensitivity analysis, we used single mean imputation to fill in missing depressive symptom data and then repeated the two‐step modelling procedure on the complete dataset.

### 2.4 Analyses of associations between depressive symptoms and PrEP adherence

We conducted two analyses of depressive symptoms and PrEP adherence. First, we estimated the association between depressive symptom trajectory group membership and PrEP adherence at Week 52 to understand how distinct depressive symptom patterns may influence PrEP use. Second, we estimated longitudinal associations between depressive symptoms and PrEP adherence to explore the effect of time‐varying symptoms on PrEP use through follow‐up.

We conducted a regression analysis, with a Poisson distribution and a log link, to model prevalence ratios between assigned group trajectory membership and detectable TFV‐DP at Week 52. We also explored associations between group membership and TFV‐DP levels ≥700 fmol/punch. Any baseline factors associated with depressive symptom trajectory membership (with *p* < 0.10) were included in the multivariable models. We included arm as a covariate to average effects across the groups with different PrEP counselling. Site was also included *a priori* to adjust for differences in demographics and sexual behaviour between locations [32, 52]. We focused only on baseline variables in this analysis for ease of interpretation and to ensure temporality.

We used generalized estimating equations to examine the association between longitudinal depressive symptoms and PrEP adherence. Models were fit with a log link, Poisson distribution and robust standard errors. Our primary model included elevated depressive symptoms as a categorical variable (CES‐D </≥10). We assessed time‐dependent covariates (e.g. sexual behaviour, IPV, stigma, social support). Any that resulted in a substantial change in the effect estimate (>10%) were included in the multivariable model. We lagged forward depressive symptom and covariate data by one visit (e.g. depressive symptoms at enrolment were used to predict PrEP adherence at Week 13) to reduce the concern of reverse temporality.

All analyses were conducted using SAS 9.4 (SAS Institute, Cary, NC, USA) and group‐based trajectory models were estimating using PROC TRAJ [51].

### 2.5 Ethical statement

This protocol was approved by ethics review committees at each sites. All participants provided written informed consent (or assent with consent from a parent or guardian if <18 years old).

## 3 Results

### 3.1 Participant characteristics

Of the 427 AGYW who initiated PrEP during the study, 33.3% were from Johannesburg, 32.8% from Cape Town and 34% from Harare (Table 1). The median age was 21 years (interquartile range [IQR]: 19 to 22; range: 16 to 25). Participants reported a median of 1 sexual partner in the three months prior to enrolment (IQR: 1 to 2), although 53 (12.4%) did not answer this question. Retention was high, with an overall retention rate of 85.4% at Week 52.

**Table 1:** HPTN 082 participant characteristics at baseline (N = 427, unless otherwise indicated)

Baseline characteristics	Frequency^a^
Arm	
Standard package	212 (49.7)
Standard package plus drug‐level feedback counselling	215 (50.4)
Study site	
Johannesburg, South Africa	142 (33.3)
Cape Town, South Africa	140 (32.8)
Harare, Zimbabwe	145 (34.0)
Age, y	21.0 (19.0 to 22.0)
Education	
Primary school	9 (2.1)
Secondary school	371 (86.9)
College or university	47 (11.0)
Number of sex partners in past three months (N = 372)	1.0 (1.0 to 2.0)
Primary sex partner in past three months (N = 425)	368 (86.6)
Number of vaginal sex acts (N = 338)^b^	4.0 (2.0 to 8.0)
Condom use with vaginal sex, past month (N = 329)^b^	
Always	68 (20.7)
Often	38 (11.6)
Sometimes	106 (32.2)
Rarely	52 (15.8)
Never	65 (19.8)
Any transactional sex in the past month (N = 425)	97 (22.8)
Elevated depressive symptoms (N = 418)^c^	179 (42.8)
Alcohol misuse (N = 419)^d^	160 (38.2)
Social support (N = 423)^e^	3.0 (2.0 to 4.0)
Support from adults in your life (N = 418)	
Almost never supported	23 (5.5)
Sometimes supported	150 (35.9)
Very well supported	245 (58.6)
Support from close friends (N = 416)	
Almost never supported	25 (6.0)
Sometimes supported	215 (51.7)
Very well supported	176 (42.3)
Stigma (N = 422)^f^	6.0 (1.0 to 7.0)
Any intimate partner violence (N = 424)^g^	209 (49.3)
Emotional intimate partner violence	157 (36.9)
Physical intimate partner violence	85 (20.0)
Sexual intimate partner violence	38 (8.9)
Ever felt afraid, unsafe, or in danger from a partner	79 (18.6)
Any posttraumatic stress disorder symptoms (N = 418)^h^	194 (46.4)

^a^ Data are presented as median (interquartile range) for continuous variables given variability and skewness observed in several of the variables. Data are presented as frequency (percentage) for categorical variables.

^b^ data on number of vaginal sex acts and condom use were only collected for participants who reported any vaginal sex in the past three months

^c^ a sum CESD‐10 score ≥10 was indicative of “elevated depressive symptoms”

^d^ an AUDIT‐C scale score ≥3 was indicative of alcohol misuse

^e^ social support was measured as the sum score across two items assessing social support from adults and close friends (range: 0 to 4)

^f^ stigma was measured as the sum score across ten items assessing stigma related to HIV and PrEP use (range: 0 to 40)

^g^ participants were considered to have experienced any intimate partner violence if they answered “yes” that they had experienced at least one of four items asking about physical, emotional, sexual violence, or feeling unsafe or in danger from a sexual partner in the past month

^h^ a response of “yes” to any of four items from the Posttraumatic Stress Disorder (PTSD) Checklist for the DSM‐5 (PCL‐5) was indicative of PTSD symptoms.

Overall, 41.9% of participants (N = 179) had CES‐D scores ≥10 at enrolment, and this proportion was lower during follow‐up with 33.0%, 36.7% and 36.9% reporting elevated depressive symptoms at Weeks 13, 26 and 52 respectively (*p*‐value for trend: 0.04; intraclass correlation coefficient: 0.39). Complete CES‐D data from all four visits were available for 320 participants. A total of 46 AGYW were missing CES‐D data at only one visit, 27 were missing data from two visits, 30 were missing data from three visits and 4 were missing from all four visits. A total of 52 AGYW (12.2%) were lost to follow‐up, defined as missing at least two CES‐D measurements without attending a subsequent visit. Missing CES‐D data were not associated with other observed data (Tables S4,S5).

### 3.2 Group‐based trajectory model

**Figure 1:**
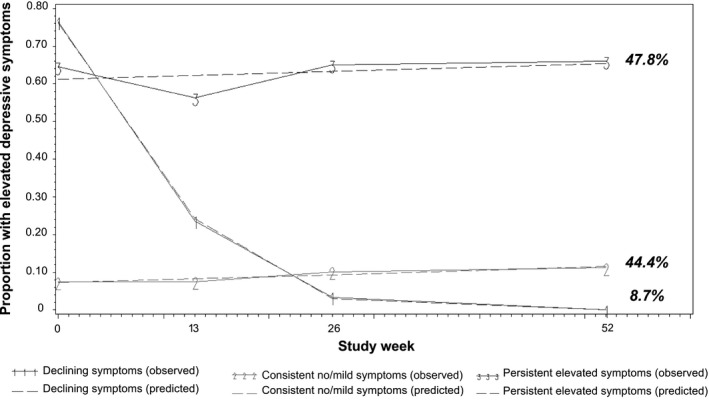
Depressive symptom trajectories for HPTN 082 participants through follow‐up (N = 423). ^*^“Observed” data points show the average proportion of participants with elevated depressive symptoms at a given study visit; “Predicted” data points show the predicted proportion with elevated depressive symptoms in each trajectory group, as estimated from a group‐based trajectory model with assigned trajectory group determined based on each participant’s highest predicted posterior group‐membership probability.

A group‐based trajectory model with three groups and linear terms had the best fit while being meaningful (Table S6). The three groups could be described as persistent elevated depressive symptoms (47.8%), declining symptoms (8.7%) and consistent no/mild symptoms (44.4%) (Figure 1). Of the 202 participants assigned to the persistent elevated symptom trajectory, 84.7% had a ≥ 0.70 posterior probability of group membership (range: 0.54 to 0.98). Of 37 participants assigned to the declining symptom trajectory, 69.9% had a ≥ 0.70 posterior probability of membership (range: 0.49 to 0.91). Of the 188 participants assigned to the consistent no/mild symptom trajectory, 83.3% had a ≥ 0.70 posterior probability of membership (range: 0.54 to 0.91). Table S7 provides additional detail on the average predicted proportions for each trajectory. Similar results were found in an exploratory analysis with a continuous CES‐D score (Figure S1) and a sensitivity analysis with imputed CES‐D data.

Any transactional sex, IPV and PTSD symptoms at baseline were associated with the depressive symptom trajectory group (Table 2). Approximately 30.5% of participants in the persistent elevated depressive symptoms group reported any transactional sex, compared with 21.6% in the declining symptom group and 14.9% in the no/mild symptom group. In the persistent elevated depressive symptoms group, 62.3% reported IPV and 58.2% had PTSD symptoms. In the declining symptoms group, 54.1% reported IPV and 51.4% had PTSD symptoms and in the no/mild symptom group, 34.6% reported IPV and 33.0% reported PTSD symptoms.

**Table 2:** Participant characteristics by depressive symptom trajectory group (N = 423)

Baseline characteristics	Frequency for each depressive symptom trajesctory^a^			*p*‐value^b^
	Consistent no/mild symptoms	Declining symptoms	Persistent elevated symptoms	
	N = 188	N = 37	N = 202	
Standard package arm	91 (48.4)	18 (48.6)	103 (51.0)	0.87
Age, years	20.0 (19.0 to 22.0)	21.0 (20.0 to 23.0)	21.0 (19.0 to 22.0)	0.18
Education				
Primary school	4 (2.1)	0 (0.0)	5 (2.5)	0.12
Secondary school	158 (84.0)	37 (100.0)	176 (87.1)	
College or university	26 (13.8)	0 (0.0)	21 (10.4)	
Number of sex partners in past three months (N = 372)	1.0 (1.0 to 2.0)	1.0 (1.0 to 1.0)	1.0 (1.0 to 2.0)	0.06
Primary sex partner in past 3 months (N = 425)	163 (86.7)	32 (86.5)	173 (86.5)	0.99
Number of vaginal sex acts (N = 338)^c^	4.0 (2.0 to 8.0)	4.0 (3.0 to 12.0)	4.0 (2.0 to 8.0)	0.92
Condom use with vaginal sex, past month (N = 329)^c^				
Always	36 (24.3)	6 (20.7)	26 (17.1)	0.06
Often	15 (10.1)	1 (3.5)	22 (14.5)	
Sometimes	49 (33.1)	6 (20.7)	51 (33.6)	
Rarely	15 (10.1)	8 (27.6)	29 (19.1)	
Never	33 (22.3)	8 (27.6)	24 (15.8)	
Any transactional sex in the past month (N = 425)	28 (14.9)	8 (21.6)	61 (30.5)	<0.01
Likely depression (N = 418)^d^	0 (0.0)	37 (100.0)	142 (72.5)	<0.01
CES‐D score^d^	5.0 (3.0 to 7.0)	12.0 (11.0 to 15.0)	11.0 (9.0 to 14.0)	<0.01
Alcohol misuse (N = 419)^e^	64 (34.2)	13 (35.1)	83 (42.6)	0.23
Social support (N = 423)^f^	3.0 (2.0 to 4.0)	3.0 (2.0 to 4.0)	3.0 (2.0 to 3.0)	0.12
Support from adults in your life (N = 418)				
Almost never supported	12 (6.5)	1 (2.7)	10 (5.1)	0.29
Sometimes supported	56 (30.4)	14 (37.8)	80 (40.6)	
Very well supported	116 (63.0)	22 (59.5)	107 (54.3)	
Support from close friends (N = 416)				
Almost never supported	7 (3.9)	2 (5.6)	16 (8.1)	0.13
Sometimes supported	87 (47.8)	22 (61.1)	106 (53.4)	
Very well supported	88 (48.4)	12 (33.3)	76 (38.4)	
Stigma (N = 422)^g^	6.0 (1.0 to 7.0)	4.0 (0.0 to 8.0)	6.0 (1.0 to 8.0)	0.53
Any intimate partner violence (N = 424)^h^	65 (34.6)	20 (54.1)	124 (62.3)	<0.01
Any emotional violence (N = 424)	50 (26.6)	16 (43.2)	91 (45.5)	0.01
Any physical violence (N = 423)	26 (13.8)	6 (16.2)	53 (26.5)	0.02
Any sexual violence (N = 423)	8 (4.3)	6 (16.2)	24 (12.0)	0.04
Ever felt afraid, unsafe, or in danger (N = 424)	16 (8.5)	11 (29.7)	52 (26.0)	<0.01
Any posttraumatic stress disorder symptoms (N = 418)^i^	61 (33.0)	19 (51.4)	224 (58.2)	<0.01

CES‐D=Center for Epidemiologic Studies – Depression Scale

^a^ Data are presented as median (interquartile range) for continuous variables and frequency (percentage) for categorical variables

^b^ *p*‐values are based on the Wilcoxon rank sum test for continuous variables and Wald *p*‐values from χ^2^ test for categorical variables

^c^ data on number of vaginal sex acts and condom use were only collected for participants who reported any vaginal sex in the past three months

^d^ a sum CESD‐10 score ≥10 was indicative of “likely depression”. CES‐D score is the sum value across ten CES‐D items (range: 0 to 30)

^e^ an AUDIT‐C scale score ≥3 was indicative of alcohol misuse

^f^ social support was measured as the sum score across two items assessing social support from adults and close friends (range: 0 to 4)

^g^ stigma was measured as the sum score across ten items assessing stigma related to HIV and PrEP use (range: 0 to 40)

^h^ participants were considered to have experienced any intimate partner violence if they answered “yes” that they had experienced at least one of four items asking about physical, emotional, sexual violence, or feeling unsafe or in danger from a sexual partner in the past month

^i^ a response of “yes” to any of four items from the Posttraumatic Stress Disorder (PTSD) Checklist for the DSM‐5 (PCL‐5) was indicative of PTSD symptoms.

### 3.3 Associations between group membership and PrEP adherence

DBS data were unavailable for 79 participants. At Week 52, 109 (31.3%) of 348 participants had detectable TFV‐DP levels. Group membership was statistically significantly associated with detectable TFV‐DP (Table 3). Participants in the persistent elevated depression symptom group were less likely to have detectable TFV‐DP levels compared with those in the consistent no/mild symptom group (adjusted prevalence ratio [aPR]: 0.89; 95% CI: 0.80 to 0.98). We were not able to explore the association between group membership and high PrEP adherence at Week 52, as only 30 participants had TFV‐DP levels ≥700 fmol/punch at the final study visit.

**Table 3:** Associations between depressive symptom trajectory membership and PrEP adherence at Week 52, assessed via multivariable regression models

Exposure group	Detectable DBS TFV‐DP levels			
	Visits with detectable TFV‐DP (N = 109/348)^b^	Adjusted GEE analysis^a^		
		aPR (95% CI)	*p*‐value	Global *p*‐value
Consistent no/mild depressive symptoms	53 (35.6%)	REF	‐‐‐	0.05
Declining depressive symptoms	12 (35.3%)	1.05 (0.46 to 2.21)	0.54	
Persistent elevated depressive symptoms	44 (26.7%)	0.89 (0.80 to 0.98)	0.04	

PrEP=pre‐exposure prophylaxis; TFV‐DP=tenofovir diphosphate; DBS=dried blood spots; aPR=adjusted prevalence ratio

^a^ Multivariable models adjusted for study site, number of sexual partners, condom use during vaginal sex, any transactional sex, any intimate partner violence and posttraumatic stress disorder symptoms, all measured at baseline

^b^ data are presented as median (interquartile range) for continuous variables and frequency (percentage) for categorical variables.

### 3.4 Associations between longitudinal depressive symptoms and PrEP adherence

TFV‐DP was detected at 607 (57.9%) follow‐up visits (intra‐class correlation coefficient: 0.46). In multivariable models adjusting for site, arm and time‐varying transactional sex, IPV, PTSD symptoms, condom use, and number of sexual partners, depressive symptoms were significantly associated with detectable TFV‐DP (adjusted relative risk [aRR]: 0.73; 95% CI: 0.53 to 0.99; Table 4). High PrEP adherence (TFV‐DP levels ≥700 fmol/punch) was detected at 193 (18.4%) of follow‐up visits. Depressive symptoms were not statistically significantly associated with high PrEP adherence (Table 4).

**Table 4:** Associations between time‐varying depressive symptoms and PrEP adherence through follow‐up, assessed via generalized estimating equations

Exposure group	Detectable DBS TFV‐DP Levels			DBS TFV‐DP ≥700 fmol/punch		
	Visits with detectable TFV‐DP (N = 607/1048)^b^	Adjusted GEE analysis^a^		Visits with TFV‐DP ≥700 fmol/punch (N = 193/1048 visits)^b^	Adjusted GEE analysis^a^	
		aRR (95% CI)	*p*‐value		aRR (95% CI)	*p*‐value
Elevated depressive symptoms (CES‐D score ≥10)	206 (55.2%)	0.73 (0.53 to 0.99)	0.04	72 (19.3%)	1.10 (0.74 to 1.62)	0.65
No/mild depressive symptoms (CES‐D score <10)	401 (59.4%)	REF	–	121 (17.9%)	REF	–

aRR, adjusted relative risk; CES‐D, Center for Epidemiologic Studies – Depression Scale; DBS, dried blood spots; GEE, generalized estimating equations; PrEP, pre‐exposure prophylaxis; TFV‐DP, tenofovir diphosphate.

^a^ Multivariable models adjusted for study site at baseline and time‐varying data on number of sexual partners, condom use during vaginal sex, any transactional sex, any intimate partner violence and posttraumatic stress disorder symptoms

^b^ data are presented as median (interquartile range) for continuous variables and frequency (percentage) for categorical variables.

## 4 Discussion

In this population of AGYW in South Africa and Zimbabwe, we identified three depressive symptom trajectory groups: persistent elevated depressive symptoms, declining symptoms and no/mild symptoms. Approximately 42% of our sample experienced elevated depressive symptoms at enrolment. About 90% had unchanging depressive symptoms, either as no/mild or persistent elevated symptoms, based on group‐based trajectory models fit with a linear functional form and CES‐D data from four time points. A smaller proportion was assigned to a trajectory with depressive symptoms that declined within the three months indicating that the study interventions, time on PrEP or other factors may have reduced depressive symptoms only for 9% of the population. Persistent elevated depressive symptom group membership and time‐varying depressive symptoms were significantly associated with detectable TFV‐DP levels.

Ours is one of the first studies to focus on depressive symptom prevalence and patterns in a population of African AGYW seeking PrEP. Other studies with African AGYW engaged in HIV treatment, other types of prevention services, or those in nationally representative cohorts report depressive symptom prevalence estimates around 40% to 50% [17, 53, 54]. Consistent with our findings, analyses of depressive symptom patterns among populations living with or at‐risk of HIV in the United States and South Africa also found distinct trajectories characterized by severe or persistent symptoms, declining symptoms and low or no symptoms [28, 29, 30, 31]. Distinct from our results, several studies also found groups characterized by worsening and recurring depressive symptoms or consistent mild‐to‐moderate symptoms [28, 29, 30, 31]. Importantly, these analyses were conducted with pregnant or recently postpartum South African adults and AGYW living with HIV in the United States. These populations likely have distinct mental health issues from African AGYW and our study adds insights on depressive symptom trajectories among African AGYW seeking PrEP. It is possible that HPTN 082 interventions may have been sufficient to prevent depressive symptoms from worsening and to address mild depressive symptoms [15, 32]. Another HIV prevention trial with women in South Africa also found relatively stable depressive symptoms through follow‐up, with about 45% of the sample reporting symptoms consistent with depression at each study visit during a six‐month period, supporting our findings on the relative stability of more moderate‐to‐severe depressive symptoms in this population [16].

We observed significant differences in depressive symptom trajectory membership by site and baseline measures of transactional sex, IPV and symptoms of PTSD. Prior studies have also reported associations between depressive symptoms, transactional sex and other HIV risk behaviour, the combination of which may contribute to co‐occurring mental health issues and HIV among AGYW [55, 56, 57]. For example recent work with 15‐ to 24‐year‐old AGYW in Kenya found that depressive symptoms were associated with transactional sex, intimate partner violence and HIV risk [58]. In HPTN 082, AGYW assigned to the persistent elevated depressive symptom trajectory group were significantly more likely to report IPV and PTSD symptoms. This may have been due to repeated experiences of IPV, difficulties accessing mental health and IPV services and unmeasured contextual factors such as lack of empowerment [59, 60, 61, 62]. Prior studies in sub‐Saharan Africa have found that IPV is associated both with a higher prevalence of depressive symptoms and PTSD symptoms, although this work has been primarily conducted with adult women [63, 64]. We did not observe differences in depressive symptom trajectory by intervention arm, possibly because participants in both arms received some PrEP adherence counselling, support groups, SMS and referrals. The drug‐level feedback counselling intervention was not targeted at reducing depressive symptoms.

We observed high drop‐offs in PrEP adherence by Week 52: only 348 (81.5%) participants had DBS samples and PrEP was detected in 31% samples at this visit. However, these results are consistent with other PrEP projects among AGYW that have reported declining PrEP adherence over time [12, 65, 66, 67]. There are several potential mechanisms to explain our findings on the significant negative relationship between depressive symptoms and PrEP use. Depression could lead to lower self‐efficacy, healthcare engagement, reduced motivation to engage in a protective health behaviour and poorer self‐care behaviour [68, 69, 70]. Persistent elevated depressive symptoms could also lead to social isolation over a prolonged period of time and less support to assist with PrEP pill‐taking [71, 72, 73]. Elevated depressive symptoms could also impact needs for PrEP, as depressive symptoms have been associated with reduced frequency of sex in some cohorts but increased condomless sex in other studies with sub‐Saharan African women [74, 75]. The impact of declining depressive symptoms on PrEP adherence is less clear. Participants in the declining depressive symptoms group had elevated depressive symptoms at enrolment which may have prevented PrEP pill‐taking habit formation early. However, it is possible that participants with declining symptoms are able to improve their PrEP use over time as their mental health improves. Future analyses with larger cohorts may be better positioned to understand this relationship between declining symptoms and PrEP adherence.

Strengths of this study include a cohort with high retention and longitudinal depressive symptom measurement using a validated screening tool. We used biomarker data on PrEP adherence rather than relying on self‐report or electronic monitoring tools. However, the small sample size, particularly among participants in the declining symptom trajectory, and drop‐off in PrEP adherence could have led to imprecise estimates of group membership and associations between symptom trajectories and PrEP adherence. The sample size and length of follow‐up may have also precluded us from identifying other trajectory groups. Approximately 12% of participants declined to answer sexual behaviour items. This may have resulted in unmeasured confounding of the relationship between depressive symptoms and PrEP adherence, although we did not detect significant associations between missing sexual behaviour data, depressive symptoms and PrEP adherence. We did not systematically collect clinical data on depression treatment and cannot make conclusions on participants’ likelihood of improving with treatment. We also did not collect data on referrals given or referral visits attended. More data on depression presentation and access to counselling and pharmacotherapy would be needed to draw further conclusions. Our group trajectories are based on CES‐D results alone and clinical evaluation was not conducted. We collected data on stigma, social support and IPV using items adapted for AGYW. However, these measures have not been widely used or validated which may have impacted the quality of construct measurement and limits comparability across populations. While we included these and other variables on sexual behaviour as covariates in our models, these factors may have a bidirectional relationship with depressive symptoms and PrEP adherence and additional research is needed to understand complex pathways between depressive symptoms, other psychosocial and behavioural factors, and PrEP use. Finally, results may not be generalizable to AGYW seeking PrEP as part of real‐world service delivery.

## 5 Conclusions

In conclusion, we found that about half of AGYW from South Africa and Zimbabwe initiating PrEP had elevated persistent depressive symptoms over one year, which was associated with lower likelihood of detectable PrEP use. Time‐varying depressive symptoms were also associated with detectable PrEP use. These findings indicate that depression assessment at PrEP initiation among African AGYW may be useful to identify women who need more intensive PrEP adherence counselling and depression treatment. While the World Health Organization currently recommends that PrEP programmes screen for mental health issues that are common causes of poor PrEP adherence [9], our results indicate that PrEP programmes could additionally screen for factors associated with persistent elevated depressive symptoms, such as transactional sex, IPV and PTSD symptoms, to target delivery of enhanced adherence support and referrals. For example AGYW with characteristics associated with persistent elevated depressive symptoms and low PrEP adherence could be linked to evidence‐based mental health and PrEP adherence interventions to increase PrEP use and reduce depressive symptoms. Evidence‐based psychotherapy, delivered alone or alongside multi‐level HIV, sexual and reproductive health, or gender‐based violence services, can improve mental health, coping around GBV and stigma and HIV‐related outcomes among African women [76, 77, 78, 79, 80, 81, 82]. Given the high prevalence of depression in this population who are at high risk of HIV acquisition, additional research is needed to adapt and implement effective depression treatment approaches in PrEP delivery for African AGYW. Identifying women most at risk for persistent depressive symptoms and poor PrEP adherence and providing targeted, integrated mental health and PrEP services could improve cost‐effective PrEP delivery for AGYW in HIV endemic settings.

## Competing interests

The authors report no conflicts of interest.

## Authors’ contributions

CC, SDM, SH, LGB, DD, NM and MC designed the parent study. JV conducted all analyses, results interpretation and wrote the first draft of the manuscript. SH, MC, NM and LGB were involved in study design, results interpretation and edited the manuscript. DD was involved in study design, statistical oversite, results interpretation and edited the manuscript. PLA was involved in study design, laboratory oversite, results interpretation and edited the manuscript. SDM and CC were involved in grant proposal development and securing funding, study design, results interpretation and edited the manuscript. All authors reviewed and approved the final version of the manuscript.

## Supporting information


**Table S1**. Social support measurement in HPTN 082
**Table S2**. Stigma measurement in HPTN 082
**Table S3**. Intimate partner violence measurement in HPTN 082
**Table S4**. Baseline characteristics for the full participant sample compared with those who had any missed CES‐D data
**Table S5**. Baseline characteristics for the full participant sample compared with those who were lost to follow‐up
**Table S6**. Group‐based trajectory model fit statistics
**Table S7**. Group‐based trajectory model output of predicted proportion with elevated depressive symptoms, by trajectory group and study visit
**Figure S1**. Depressive symptom trajectories for HPTN 082 participants through follow‐up, using continuous CES‐D score
