# Supplementary material for: Assessing longitudinal patterns of depressive symptoms and the influence of symptom trajectories on HIV pre‐exposure prophylaxis adherence among adolescent girls in the HPTN 082 randomized controlled trial
Source: J Int AIDS Soc. 2021 Jun 24;24(Suppl 2):e25731. doi: 10.1002/jia2.25731 (PMC8222844; doi:10.1002/jia2.25731)
Supplement: Supplementary file 1 — Table S1. Social support measurement in HPTN 082 Table S2. Stigma measurement in HPTN 082 Table S3. Intimate partner violence measurement in HPTN 082 Table S4. Baseline characteristics for the full participant sample compared with those who had any missed CES‐D data Table S5. Baseline characteristics for the full participant sample compared with those who were lost to follow‐up Table S6. Group‐based trajectory model fit statistics Table S7. Group‐based trajectory model output of predicted proportion with elevated depressive symptoms, by trajectory group and study visit Figure S1. Depressive symptom trajectories for HPTN 082 participants through follow‐up, using continuous CES‐D score [file JIA2-24-e25731-s001.docx]

**APPENDIX.** Supplemental information on data collection measures and analyses

**Table A1.** Social support measurement in HPTN 082**V: STIGMA**

| **Show if: VISIT = 06.0 OR 07.0 OR ((VISIT = 16.0, 17.0, OR 52.0) AND PREPACCEPT = 0/No)**  In general, how supported do you feel by the adults in your life? **V: STASHAMED** | 1 = Almost never supported | 2 = Sometimes supported | 3 = Almost always supported |
| --- | --- | --- | --- |
| **Show if: VISIT = 06.0 OR 07.0 OR ((VISIT = 16.0, 17.0, OR 52.0) AND PREPACCEPT = 0/No)**  In general, how supported do you feel by your close friends?**V: STEMBARR** | 1 = Almost never supported | 2 = Sometimes supported | 3 = Almost always supported |

**Table A2.** Stigma measurement in HPTN 082**V: STIGMA**

| **Show if: VISIT = 06.0 OR 07.0 OR ((VISIT = 16.0, 17.0, OR 52.0) AND PREPACCEPT = 0/No)**  I feel ashamed of using PrEP. **V: STASHAMED** | 1 = Strongly Disagree | 2 = Disagree | 3 = Neither Agree nor Disagree | 4 = Agree | 5 =Strongly Agree | 99 = Prefer not to answer |
| --- | --- | --- | --- | --- | --- | --- |
| **Show if: VISIT = 06.0 OR 07.0 OR ((VISIT = 16.0, 17.0, OR 52.0) AND PREPACCEPT = 0/No)**  I feel embarrassed about using PrEP. **V: STEMBARR** | 1 = Strongly Disagree | 2 = Disagree | 3 = Neither Agree nor Disagree | 4 = Agree | 5 =Strongly Agree | 99 = Prefer not to answer |
| **Show if: VISIT = 06.0 OR 07.0 OR ((VISIT = 16.0, 17.0, OR 52.0) AND PREPACCEPT = 0/No)**  I think I am not following the ‘rules' of my community if I take PrEP to prevent HIV. **V: STRULES** | 1 = Strongly Disagree | 2 = Disagree | 3 = Neither Agree nor Disagree | 4 = Agree | 5 =Strongly Agree | 99 = Prefer not to answer |
| **Show if: VISIT = 06.0 OR 07.0 OR ((VISIT = 16.0, 17.0, OR 52.0) AND PREPACCEPT = 0/No)**  I think people will give me a hard time if I tell them I am on PrEP. **V: STHARDTM** | 1 = Strongly Disagree | 2 = Disagree | 3 = Neither Agree nor Disagree | 4 = Agree | 5 =Strongly Agree | 99 = Prefer not to answer |
| **Show if: VISIT = 06.0 OR 07.0 OR ((VISIT = 16.0, 17.0, OR 52.0) AND PREPACCEPT = 0/No)**  I think people will judge me if I am taking PrEP. **V: STJUDGE** | 1 = Strongly Disagree | 2 = Disagree | 3 = Neither Agree nor Disagree | 4 = Agree | 5 =Strongly Agree | 99 = Prefer not to answer |
| **Show if: VISIT = 06.0 OR 07.0 OR ((VISIT = 16.0, 17.0, OR 52.0) AND PREPACCEPT = 0/No)**  I think I am at greater risk for physical violence or rape if I am taking PrEP **V: STVIOLNC** | 1 = Strongly Disagree | 2 = Disagree | 3 = Neither Agree nor Disagree | 4 = Agree | 5 =Strongly Agree | 99 = Prefer not to answer |

**Table A3.** Intimate partner violence measurement in HPTN 082

| Script: Now we will ask you some questions about your relationships with your partner, family, friends, or other people in your neighborhood. We know that relationships can have good and bad moments. Some questions may be difficult to answer and we would like to remind you that your answers will be kept confidential. | | | |
| --- | --- | --- | --- |
| In the past month, has your current or most recent partner punched, slapped, kicked, bit you, or caused you any time of physical harm? | Yes | No | Prefer not to answer |
| In the past month, has your current or most recent partner insulted, ignored or humiliated you, yelled at you, or made you feel ashamed or bad about yourself? | Yes | No | Prefer not to answer |
| In the past month, has your current or most recent partner forced you to have sex or perform any sexual act, or touched you sexually in any way that you did not want? | Yes | No | Prefer not to answer |
| In the past month, has your current or most recent partner made you feel afraid, unsafe, or in danger? | Yes | No | Prefer not to answer |

**Table A4.** Baseline characteristics for the full participant sample compared with those who had any missed CES-D data

| **Baseline characteristics** | **Frequency^1^** | | **p-value** |
| --- | --- | --- | --- |
|  | **Participants with full CES-D data (N=320)** | **Participants with any missing CES-D data (N=107)** |  |
| Arm  Standard package  Standard package plus DLFB | 161 (50.3)  159 (50.0) | 51 (47.7)  567 (52.3) | 0.64 |
| Study site  Johannesburg, South Africa  Cape Town, South Africa  Harare, Zimbabwe | 97 (30.3)  112 (35.0) 111 (34.7) | 45 (42.1)  28 (26.2)  34 (31.8) | 0.07 |
| Age, years | 21 (19-22) | 21 (19-22) | 0.53 |
| Education  Primary school  Secondary school  College or university | 7 (2.2)  279 (87.2)  34 (10.6) | 2 (1.9)  92 (86.0)  13 (12.2) | 0.90 |
| Number of sex partners, past 3 months | 1 (1-2) | 1 (1-2) | 0.99 |
| Number of vaginal sex acts, past 3 months | 4 (3-9) | 3 (2-5.5) | 0.16 |
| Condom use with vaginal sex, past month^2^  Always  Often  Sometimes  Rarely  Never | 44 (18.3)  28 (11.6)  82 (34.0)  40 (16.6)  47 (19.5) | 24 (27.3)  10 (11.4)  24 (27.3)  12 (13.6)  19 (20.5) | 0.43 |
| Any transactional sex in the past month | 72 (22.5) | 25 (23.8) | 0.78 |
| Elevated depressive symptoms^3^ | 138 (43.1) | 41 (41.8) | 0.82 |
| Alcohol misuse^4^ | 122 (38.5) | 38 (37.3) | 0.82 |
| Social support^5^ | 3 (2-4) | 3 (2-4) | 0.27 |
| Stigma^6^ | 6 (1-7) | 6 (1-8) | 0.48 |
| Any intimate partner violence^7^ | 165 (51.6) | 44 (42.3) | 0.10 |
| Any posttraumatic stress disorder symptoms^8^ | 141 (44.3) | 53 (53.0) | 0.13 |

DLFB=drug-level feedback counseling

^1^Data are presented as median (interquartile range) for continuous variables given variability and skewness observed in several of the variables. Data are presented as frequency (percentage) for categorical variables.

^2^Data on number of vaginal sex acts and condom use were only collected for participants who reported any vaginal sex in the past three months

^3^A sum CESD-10 score ≥10 was indicative of “elevated depressive symptoms”

^4^An AUDIT-C scale score ≥3 was indicative of alcohol misuse

^5^Social support was measured as the sum score across two items assessing social support from adults and close friends (range: 0-4)

^6^Stigma was measured as the sum score across ten items assessing stigma related to HIV and PrEP use (range: 0-40)

^7^Participants were considered to have experienced any intimate partner violence if they answered “yes” that they had experienced at least one of four items asking about physical, emotional, sexual violence, or feeling unsafe or in danger from a sexual partner in the past month

^8^A response of “yes” to any of four items from the Posttraumatic Stress Disorder (PTSD) Checklist for the DSM-5 (PCL-5) was indicative of PTSD symptoms

**Table A5.** Baseline characteristics for the full participant sample compared with those who were lost to follow-up

| **Baseline characteristics** | **Frequency^1^** | | **p-value** |
| --- | --- | --- | --- |
|  | **Retained participants (N=375)** | **Participants lost to follow-up (N=52)^2^** |  |
| Arm  Standard package  Standard package plus DLFB | 187 (50.0)  188 (50.1) | 25 (48.1)  27 (51.9) | 0.81 |
| Study site  Johannesburg, South Africa  Cape Town, South Africa  Harare, Zimbabwe | 120 (32.0)  128 (34.1)  127 (33.9) | 22 (42.3)  12 (23.1)  18 (34.6) | 0.21 |
| Age, years | 21 (19-22) | 21 (20-22) | 0.38 |
| Education  Primary school  Secondary school  College or university | 8 (2.1)  326 (86.9)  41 (10.9) | 1 (1.9)  45 (86.5)  6 (11.5) | 0.99 |
| Number of sex partners, past 3 months | 1 (1-2) | 1 (1-2) | 0.99 |
| Number of vaginal sex acts, past 3 months | 4 (3-8) | 3 (2-5.5) | 0.53 |
| Condom use with vaginal sex, past month^3^  Always  Often  Sometimes  Rarely  Never | 57 (20.0)  33 (11.6)  93 (32.6)  46 (16.1)  56 (19.7) | 11 (25.0)  5 (11.4)  13 (29.6)  6 (13.6)  9 (20.5) | 0.95 |
| Any transactional sex in the past month | 84 (22.5) | 13 (25.0) | 0.69 |
| Elevated depressive symptoms^4^ | 160 (43.2) | 19 (39.6) | 0.63 |
| Alcohol misuse^5^ | 145 (39.3) | 15 (30.0) | 0.20 |
| Social support^6^ | 3 (2-4) | 3 (2-4) | 0.95 |
| Stigma^7^ | 6 (1-7) | 6 (1-8) | 0.54 |
| Any intimate partner violence^8^ | 188 (50.4) | 21 (41.2) | 0.22 |
| Any posttraumatic stress disorder symptoms^9^ | 169 (45.9) | 25 (50.0) | 0.59 |

DLFB=drug-level feedback counseling

^1^Data are presented as median (interquartile range) for continuous variables given variability and skewness observed in several of the variables. Data are presented as frequency (percentage) for categorical variables.

^2^Loss to follow-up defined as having missing CES-D data for at least two consecutive study visits without attending a subsequent visit.

^3^Data on number of vaginal sex acts and condom use were only collected for participants who reported any vaginal sex in the past three months

^4^A sum CESD-10 score ≥10 was indicative of “elevated depressive symptoms”

^5^An AUDIT-C scale score ≥3 was indicative of alcohol misuse

^6^Social support was measured as the sum score across two items assessing social support from adults and close friends (range: 0-4)

^7^Stigma was measured as the sum score across ten items assessing stigma related to HIV and PrEP use (range: 0-40)

^8^Participants were considered to have experienced any intimate partner violence if they answered “yes” that they had experienced at least one of four items asking about physical, emotional, sexual violence, or feeling unsafe or in danger from a sexual partner in the past month

^9^A response of “yes” to any of four items from the Posttraumatic Stress Disorder (PTSD) Checklist for the DSM-5 (PCL-5) was indicative of PTSD symptoms

**Table A6.** Group-based trajectory model fit statistics

| **Step 1: Comparing number of trajectory groups** | | | | | | |
| --- | --- | --- | --- | --- | --- | --- |
| N trajectories | BIC (N=1502)^1^ | BIC (N=423)^2^ | AIC | LL | Smallest group % | Average posterior probability, per group |
| 2 | -4563.86 | -4560.06 | -4547.91 | -4541.91 | 38.7 | 0.88, 0.85 |
| *3* | *-4552.13* | *-4546.43* | *-4528.22* | *-4519.22* | *12.2* | *0.84, 0.78, 0.83* |
| 4 | -4552.88 | -4545.28 | -4520.99 | -4508.99 | 1.9 | 0.86, 0.84, 0.66, 0.82 |
| 5 | -4558.00 | -4548.50 | -4518.15 | -4503.15 | 1.9 | 0.88, 0.85, 0.71, 0.76, 0.77 |
| 6 | -4568.98 | -4557.57 | -4521.15 | -4503.15 | 0.0 | 0.88, 0.00, 0.85, 0.71, 0.76, 0.77 |
| **Step 2: Comparing functional form of trajectory groups** | | | | | | |
| Shape parameter^3^ | BIC (N=1502)^1^ | BIC (N=423)^2^ | AIC | LL | Smallest group % | Average posterior probability, per group |
| *1, 1, 1* | *-4552.13* | *-4546.43* | *-4528.22* | *-4519.22* | *12.2* | *0.84, 0.78, 0.83* |
| 1, 2, 2 | -4557.17 | -4550.20 | -4527.94 | -4516.94 | 8.2 | 0.84,0.80, 0.86 |
| 2, 2, 2 | -4556.46 | -4548.85 | -4524.57 | -4512.57 | 8.1 | 0.84, 0.81, 0.86 |
| 2, 2, 3 | -4552.94 | -4544.71 | -4518.40 | -4505.40 | 9.1 | 0.85, 0.81, 0.85 |
| 2, 3, 3 | -4556.03 | -4547.16 | -4518.83 | -4504.83 | 9.5 | 0.85, 0.81, 0.86 |
| 3, 3, 3 | -4559.69 | -4550.18 | -4519.83 | -4504.83 | 9.5 | 0.85, 0.81, 0.86 |

BIC=Bayesian Information Criteria; AIC=Akaike Information Criteria; LL=Log likelihood

Italics indicates selected model

^1^N is number of observations included in the model

^2^N is number of participants included in the model

^3^Shape parameter of 1=linear, 2=quadratic, 3=cubic functional forms

**Table A7.** Group-based trajectory model output of predicted proportion with elevated depressive symptoms, by trajectory group and study visit

| **Study Visit** | **Predicted proportion of participants with elevated depressive symptoms (95% CI)** | | |
| --- | --- | --- | --- |
|  | Declining symptom trajectory | Consistent no/mild symptoms trajectory | Persistent elevated symptoms |
| Enrollment | 0.76 (0.56-1.00) | 0.07 (0.00-0.17) | 0.64 (0.53-0.69) |
| Week 13 | 0.24 (0.00-0.53) | 0.07 (0.00-0.16) | 0.56 (0.50-0.69) |
| Week 26 | 0.04 (0.00-0.24) | 0.10 (0.03-0.15) | 0.65 (0.56-0.70) |
| Week 52 | 0.00 (0.00-0.01) | 0.11 (0.01-0.22) | 0.66 (0.54-0.77) |

95% CI=95% confidence interval

**Figure A1.** Depressive symptom trajectories for HPTN 082 participants through follow-up, using continuous CES-D score

**
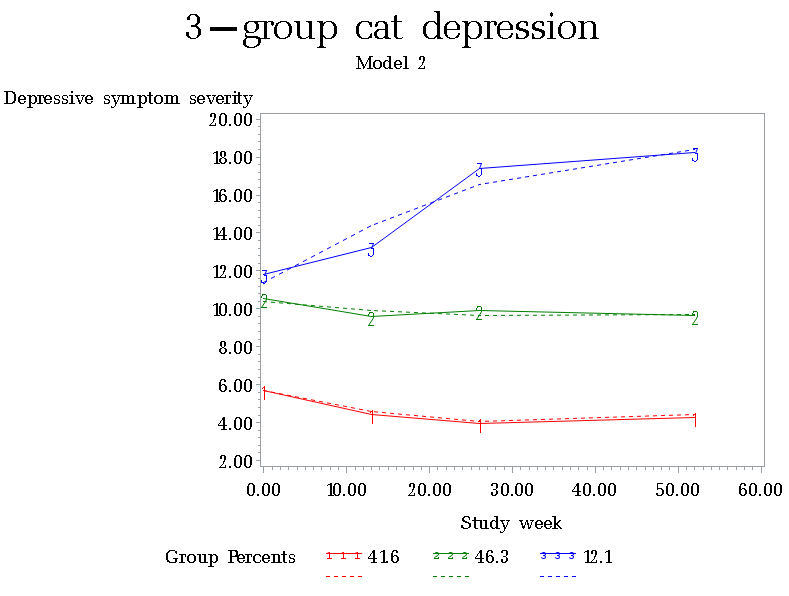

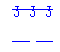

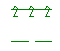

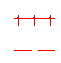
**

*41.6%*

*46.3%*

*12.1%*

Persistent elevated symptoms (predicted)

Persistent elevated symptoms (observed)

Consistent mild/moderate symptoms (predicted)

Consistent mild/moderate symptoms (observed)

Consistent low/no symptoms (predicted)

Consistent low/no symptoms (observed)
